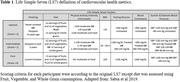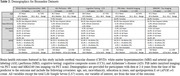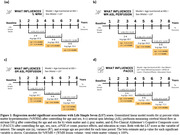# Evaluating Life Simple Seven’s Influence on Brain Health Outcomes: The Intersection of Lifestyle and Dementia

**DOI:** 10.1002/alz.091720

**Published:** 2025-01-09

**Authors:** Diandra N. Denier‐Fields, Ronald E. Gagnon, Brenda Ryther, Ian A. Canovi, Diane Wilkinson, Lia J. Sparks, Leonardo A. Rivera‐Rivera, Tobey J. Betthauser, Barbara B. Bendlin, Sterling C. Johnson, Corinne D. Engelman

**Affiliations:** ^1^ University of Wisconsin‐Madison, Madison, WI USA; ^2^ Wisconsin Alzheimer’s Institute, University of Wisconsin‐Madison School of Medicine and Public Health, Madison, WI USA; ^3^ Wisconsin Alzheimer’s Institute, University of Wisconsin‐Madison, Madison, WI USA; ^4^ Department of Medicine, University of Wisconsin‐Madison School of Medicine and Public Health, Madison, WI USA; ^5^ School of Medicine and Public Health, University of Wisconsin‐Madison, Madison, WI USA; ^6^ Wisconsin Alzheimer’s Disease Research Center, Madison, WI USA; ^7^ Alzheimer’s Disease Research Center, University of Wisconsin‐Madison, Madison, WI USA; ^8^ Geriatric Research Education and Clinical Center, William S. Middleton Memorial Veterans Hospital, Madison, WI USA; ^9^ Wisconsin Alzheimer’s Institute, University of Wisconsin School of Medicine and Public Health, Madison, WI USA

## Abstract

**Background:**

Lifestyle factors have been studied for their role in dementia, but few have comprehensively assessed both Alzheimer’s disease (AD) and cerebrovascular disease (CBVD) pathologies. This study innovatively integrates AD, CBVD, and cognitive composite scores (CCS) within the same cohort to investigate the association of the Life Simple Seven (LS7) score with a broad spectrum of dementia‐related outcomes. Our research aims to unravel the intricate relationships between lifestyle and various dementia pathologies, challenging conventional research paradigms.

**Method:**

Analyzing 1210 Wisconsin Registry for Alzheimer’s Prevention (WRAP) study participants, we focused on LS7 score calculations from questionnaire data and clinical vitals (Table 1). We assessed brain health indicators over intervals of 2‐4, 4‐6, and 6‐8 years post‐LS7 scoring. CBVD outcomes included white matter hyperintensities (WMH) and cerebral blood flow (CBF); CCS outcomes comprised delayed recall, executive function, immediate learning, and the Pre‐Clinical Alzheimer Cognitive Composite 3 (PACC3); AD outcomes involved PET amyloid (PiB) and tau (MK6240) imaging (Table 2). Our study controlled for age, sex, race/ethnicity, apolipoprotein E ε4 (*APOE* ε4) count, years of education, and practice effects as appropriate for each outcome.

**Result:**

Higher LS7 scores were associated with 0.06 lower %WMH at 2‐4 years, per point increase in LS7 score (n = 275, p = 0.005), contrasted with 0.05 higher %WMH annually for participants over 65 (p<0.001; Figure 1). In relation to CBF, although there is a general decrease during aging that accelerates in disease states, higher LS7 scores were associated with relatively higher ASL perfusion in both gray and white matter at 2‐4 and 6‐8 years. For PACC3 scores, one point increase in LS7 score was associated with 0.04 higher scores (n = 872, p = 0.041) at the 2‐4 year interval, while an increase in age was associated with lower PACC3 scores by 0.05 (p≤0.001). No significant associations were found between LS7 scores and the remaining CCS or AD imaging.

**Conclusion:**

This study highlights the impact of cardiovascular health, influenced by lifestyle choices, on cognition, likely through the cerebrovascular pathway. It underscores the importance of lifestyle interventions in vascular dementia prevention and management, offering critical insights for developing strategies to mitigate cognitive decline and dementia risk.